# The best triathletes are older in longer race distances – a comparison between Olympic, Half-Ironman and Ironman distance triathlon

**DOI:** 10.1186/2193-1801-3-538

**Published:** 2014-09-18

**Authors:** Raphael Knechtle, Christoph Alexander Rüst, Thomas Rosemann, Beat Knechtle

**Affiliations:** Institute of Primary Care, University of Zurich, Zurich, Switzerland; Gesundheitszentrum St. Gallen, Vadianstrasse 26, 9001 St. Gallen, Switzerland

**Keywords:** Age trends, Endurance, Swimming, Cycling, Running

## Abstract

The purpose of this study was (*i*) to determine the age of peak triathlon performance for world class athletes competing in Olympic, Half-Ironman and Ironman distance races and (*ii*) to investigate a potential change in the age of the annual fastest athletes across years. Data of ages and race times of all finishers in the international top races over the three distances between 2003 and 2013 were collected and the annual top ten women and men were analysed using linear, non-linear and hierarchical multivariate regression analyses. The age of peak male performance was 27.1 ± 4.9 years in the Olympic, 28.0 ± 3.8 years in the Half-Ironman and 35.1 ± 3.6 years in the Ironman distance and the age of peak male performance was higher in the Ironman compared to the Olympic (*p* < 0.05) and the Half-Ironman distance (*p* < 0.05) triathlon. The age of peak female performance was 26.6 ± 4.4 years in the Olympic, 31.6 ± 3.4 years in the Half-Ironman and 34.4 ± 4.4 years in the Ironman distance and the age of peak female performance was lower in the Olympic compared to the Half-Ironman (*p* < 0.05) and Ironman distance (*p* < 0.05) triathlon. The age of the annual top ten women and men remained unchanged over the last decade in the Half-Ironman and the Ironman distance. In the Olympic distance, however, the age of the annual top ten men decreased slightly. To summarize, the age of peak triathlon performance was higher in the longer triathlon race distances (*i.e.* Ironman) and the age of the annual top triathletes remained mainly stable over the last decade. With these findings top athletes competing at world class level can plan their career more precisely as they are able to determine the right time in life to switch from the shorter (*i.e.* Olympic distance) to the longer triathlon race distances (*i.e.* Half-Ironman and Ironman) in order to continuously compete in triathlon races at world class level.

## Background

Triathlon is a unique endurance sport including the three disciplines swimming, cycling and running in this order. Triathlon races are held over different distances such as the Olympic or short distance triathlon (*i.e.* 1.5 km swimming, 40 km cycling and 10 km running), the Half-Ironman or Ironman 70.3 distance (*i.e.* 1.9 km swimming, 90 km cycling and 21.1 km running) and the Ironman distance (*i.e.* 3.8 km swimming, 180 km cycling and 42.2 km running) (Bentley et al. 2002). In addition to the Ironman triathlon distance, ultra-endurance triathlons of longer distances do exist, such as the Triple Iron ultra-triathlon (*i.e.* 11.4 km swimming, 540 km cycling and 126.6 km running) (Knechtle et al. 2008), and the Deca Iron ultra-triathlon (*i.e.* 38 km swimming, 1,800 km cycling and 420 km running) (Herbst et al. 2011).

Over the last decade, several studies showed an age-related decline in endurance performance (Bernard et al. 2010; Knechtle et al. 2012c; Ransdell et al. 2009. Tanaka and Seals 2008). These studies demonstrated that endurance performance appeared to be maintained until the age of ~35-40 years, with a modest decrease until the age of ~50 years, followed by a progressive decrease in performance thereafter (Reaburn and Dascombe 2008; Sultana et al. 2008; Tanaka and Seals 2008). After the age of ~70 years, the greatest declines in endurance performance occur (Lepers et al. 2013a). Triathlon represents an interesting model to analyse the age-related decline of endurance performances in both recreational and elite athletes, as the decline in performance can be analysed in the same individual for the three disciplines separately (Bernard et al. 2010; Lepers and Maffiuletti 2011).

Considering Olympic and Ironman distance triathlon, overall race times decreased progressively with advancing age, but there was a smaller age-related decline in the cycling split performance than in the running and the swimming split performances (Lepers et al. 2010). This means that the age-related decline in triathlon performance differs between the locomotion modes (Bentley et al. 2002; Knechtle et al. 2012a). Physiological and mechanical specificities of cycling compared to running and swimming, such as the change from a non-weight to a weight-bearing activity and the shift from a stretch-shortening activity with eccentric contractions in running to a concentric type of muscle action in cycling (Bijker et al. 2002), and different training stimulus may explain the lower age-related performance decline in cycling compared to running and swimming (Bentley et al. 2002; Lepers et al. 2010).

The decline in triathlon performance with increasing age has been investigated for split times in the Olympic distance (Bentley et al. 2002), the Half-Ironman distance (Knechtle et al. 2012b) and the Ironman distance (Lepers et al. 2013b; Stiefel et al. 2012) triathlon. Interestingly, the age-related performance decline started at different ages in the three different race distances. In the Olympic distance, the decline in performance for split times started at the age of ~40 years for swimming, at ~50 years for running and at ~55 years for cycling (Lepers et al. 2010). In the Half-Ironman distance, the decline started at the age of ~40 years for both swimming and cycling, whereas the decline in running performance started at the age of ~45 years (Knechtle et al. 2012b). In the Ironman distance, the performance started to decrease at the age of ~45 years for swimming and running, and at the age of ~50 years for cycling (Lepers et al. 2010). The reason for the earlier decline in running performance compared to cycling performance might be due to the differences in locomotion between cycling and running which are the change from a non-weight-bearing to a weight-bearing activity and the shift from a stretch-shortening activity with eccentric contractions in running to a concentric type of muscle action in cycling (Bijker et al. 2002). Since different rates of decline during fatiguing contractions involving eccentric compared to concentric activations occur, this might explain the different rate of decline in cycling compared to running (Heiden and Burnett 2003).

The age-related decline of endurance performance over the different distances in triathlon has been well examined and differs between the different race distances (Bentley et al. 2002; Knechtle et al. 2012b; Lepers et al. 2010; 2013b; Stiefel et al. 2012). However, the exact age of peak triathlon performance has not been investigated yet for all triathlon distances. Up to now, no study has investigated at which age the athletes achieve their peak triathlon performance in the Olympic distance. Women competing in a Half-Ironman such as the ‘Ironman 70.3 Switzerland’ race, a qualifier for the Ironman World Championship 70.3, achieved their peak triathlon performance between the age of ~25 and ~39 years and men between ~18 and ~39 years (Knechtle et al. 2012b). The fastest race times in ‘Ironman Hawaii’ were achieved by women in age groups 25–29 and 30–34 years, and men in age groups 30–34 and 35–39 years (Lepers and Maffiuletti 2011). It has been reported that the age of peak triathlon performance in Ironman triathlon was at ~33-34 years for both women and men (Gallmann et al. 2014; Rüst et al. 2012a; Stiefel et al. 2013a). For longer distances than the Ironman distance, Knechtle et al. (2012a) showed that the mean age of male winning athletes in a Triple Iron ultra-triathlon was ~36 years and the mean age of male winning athletes in a Deca Iron ultra-triathlon was ~38 years.

Several studies investigating participation trends of master athletes showed an increase in participation and an improvement of triathlon performance of master athletes in recent years, while the performances of athletes younger than 40 years remained quite stable (Etter et al. 2013; Gallmann et al. 2014; Lepers et al. 2013b; Stiefel et al. 2012). During the 1986–2010 period in ‘Ironman Hawaii’ as the official Ironman World Championship, men older than 44 years and women older than 40 years significantly improved their performance in both the split disciplines and in overall race times (Lepers et al. 2013b). The age of the annual ten fastest women and men in ‘Ironman Hawaii’ increased over the last three decades and their performance improved while younger athletes seemed to have reached their limits in Ironman triathlon performance (Gallmann et al. 2014; Lepers et al. 2013b).

The age of peak triathlon performance was higher in athletes competing in the Ironman distance (Stiefel et al. 2013a) compared to the Half-Ironman distance triathlon (Knechtle et al. 2012b), and the decline of performance started earlier in the Olympic distance compared to the longer distances (Etter et al. 2013, Lepers et al. 2010). Considering longer distances, the age of peak triathlon performance was higher in athletes competing in the Triple Iron and the Deca Iron ultra-triathlon than for shorter triathlon distances, leading to the assumption that the age of peak triathlon performance would be higher in longer triathlon races (*i.e.* Half-Ironman and Ironman distance) compared to shorter races (*i.e.* Olympic distance) (Knechtle et al. 2012a). The age of peak triathlon performance in Olympic distance triathlon has not previously been investigated and up to now, only age-group Half-Ironman performances have been analyzed. Generally, there are fewer studies investigating the age of peak triathlon performance in longer triathlon races (Knechtle et al. 2012b; Rüst et al. 2012a; Stiefel et al. 2013a) than studies investigating the age-related decline in triathlon performance (Bernard et al. 2010; Etter et al. 2013; Lepers et al. 2010; 2013a; [b]; Stiefel et al. 2012).

For athletes and their coaches, the age of peak triathlon performance is more important than the age-related decline in performance (Lepers et al. 2013a). With the knowledge of the exact age of peak triathlon performance for the different distances, athletes would be able to determine the best time in life to race in the different distances. Due to the gap in the literature about the exact age of peak triathlon performance over the different distances, athletes may not have been able to organize their training and plan their career as precisely as they could have with these findings. Therefore, the first aim of this study was to determine the age of peak triathlon performance for world class athletes competing in the Olympic distance using race data of the top international races of the last decade. Since the age of peak triathlon performance appears to increase with increasing race distance, we expected the age of peak triathlon performance for the Olympic distance to be lower than for the Half-Ironman and Ironman distances. The second aim was to determine the age of peak triathlon performance for world class athletes competing in the Half-Ironman and the Ironman distances and it was assumed that the age of peak triathlon performance would be higher in the longer race distances (*i.e.* Half-Ironman and Ironman). We expected that the age of peak triathlon performance would be higher in the Half-Ironman distance than in the Olympic distance, and higher in the Ironman distance than in both the Olympic and the Half-Ironman distances. The third aim was to investigate a potential change in the age of peak triathlon performance for the annual top athletes between 2003 and 2013 for the three different distances. It has been reported that the fastest athletes competing in ‘Ironman Hawaii’ became older and faster over the past few years (Gallmann et al. 2014). We expected that the age of peak triathlon performance for the fastest Ironman triathletes would increase over the last decade, while it would remain stable in athletes competing in the Olympic and Half-Ironman distance. The forth aim of this study was to investigate potential changes in triathlon performance over time. Since athletes competing in ‘Ironman Hawaii’ became faster over the past few years (Gallmann et al. 2014), we expected to find an improvement of performance in the Ironman distance, while performance would remain stable in the Olympic and Half-Ironman distance.

## Methods

### Ethics

The study was approved by the Institutional Review Board of St. Gallen, Switzerland, with a waiver of the requirement for informed consent given that the study involved the analysis of publicly available data.

### Data sampling and data analysis

The data were obtained from the websites of the ITU (http://www.triathlon.org/results) for Olympic distance races and for Half-Ironman and Ironman distance races from the official site of Ironman triathlon races (http://eu.ironman.com/#axzz2qO2hTgJf). The races from which the data was obtained were chosen with regards to the highest competition level (*i.e.* world class level). Therefore, all the included races were either World Championship or Continental Championship races. In order to be able to compete at these events athletes need to qualify in qualifier races leading to a high level in competition. All female and male triathletes who finished the top international races in the ITU (International Triathlon Union) World Triathlon Series races and in both the World and Continental Championship races over the three different distances between 2003 and 2013 in the top ten were analysed for race distance, performance and age.

For the Olympic distance (*i.e.* 1.5 km swimming, 40 km cycling and 10 km running), overall race times and split times of the top ten male and female athletes competing in 38 ITU World Triathlon Series races from 2009 to 2013 were collected (Table 1), since the World Championship in triathlon has been replaced by the ITU World Triathlon Series in 2009. Additionally to this data, overall race times and split times of the top ten women and men competing in six World Championship races from 2003 to 2008 were obtained. For the Half-Ironman distance (*i.e.* 1.9 km swimming, 90 km cycling and 21.1 km running), overall race times and split times of the top ten women and men competing in eight World Championship races from 2006 to 2013 and eleven Continental Championships from 2007 to 2013 were collected (Table 2). For the Ironman distance (*i.e.* 3.8 km swimming, 180 km cycling and 42.2 km running), split and overall race times of the top ten women and men competing in eleven World Championship races from 2003 to 2013 and twelve Continental Championship races from 2005 to 2013 were collected (Table 3). Transition times between swimming and cycling and between cycling and running were included in the overall race time for all the collected data.

**Table 1:** **Number of ITU elite male and female starters and finishers in Olympic distance races from 2003 to 2013, WC = World Championship, ITU = International Triathlon Union**

Race	Date	Location	Women finished	Women started	Men finished	Men started
WC	11-09-2013	London	47	54	60	67
ITU	24-08-2013	Stockholm	40	45	43	58
ITU	20-07-2013	Hamburg	61	65	58	65
ITU	06-07-2013	Kitzbuehel	38	39	62	65
ITU	01-06-2013	Madrid	40	53	47	62
ITU	11-05-2013	Yokohama	21	22	35	38
ITU	19-04-2013	San Diego	36	40	46	55
ITU	06-04-2013	Auckland	24	31	28	33
WC	20-10-2012	Auckland	33	43	39	44
ITU	29-09-2012	Yokohama	28	33	35	39
ITU	25-08-2012	Stockholm	51	57	58	65
ITU	21-07-2012	Hamburg	46	50	59	61
ITU	23-06-2012	Kitzbuehel	30	39	46	53
ITU	26-05-2012	Madrid	55	63	54	65
ITU	10-05-2012	San Diego	58	70	61	70
ITU	14-04-2012	Sydney	60	68	57	70
WC	19-09-2011	Yokohama	46	55	52	65
ITU	09-09-2011	Beijing	58	69	59	72
ITU	06-08-2011	London	56	65	59	65
ITU	16-07-2011	Hamburg	51	59	50	63
ITU	18-06-2011	Kitzbuehel	58	65	52	64
ITU	04-06-2011	Madrid	53	65	58	65
ITU	09-04-2011	Sydney	56	56	52	65
WC	08-09-2010	Budapest	56	68	66	75
ITU	14-08-2010	Kitzbuehel	48	64	52	65
ITU	24-07-2010	London	50	65	56	65
ITU	17-07-2010	Hamburg	52	64	53	65
ITU	05-06-2010	Madrid	56	67	54	54
ITU	08-05-2010	Seoul	42	50	55	68
ITU	11-04-2010	Sydney	39	45	46	55
WC	09-09-2009	Gold Coast	37	42	51	53
ITU	22-08-2009	Yokohama	28	30	36	44
ITU	15-08-2009	London	47	58	47	65
ITU	25-07-2009	Hamburg	37	46	51	62
ITU	11-07-2009	Kitzbuehel	40	43	49	66
ITU	21-06-2009	Washington	30	38	31	46
ITU	31-05-2009	Madrid	35	47	41	60
ITU	02-05-2009	Tongyeong	41	48	66	69
WC	08-06-2008	Vancouver	51	67	71	79
WC	01-09-2007	Hamburg	60	77	68	80
WC	03-09-2006	Lausanne	64	76	68	86
WC	11-09-2005	Gamagori	43	51	55	77
WC	09-05-2004	Madeira	57	74	72	89
WC	07-12-2003	Queenstown	52	61	57	75

**Table 2:** **Number of male and female pro finishers in Half-Ironman races from 2003 to 2013, WC = World Championship, CC = Continental Championship**

Race	Date	Location	Women finished	Women started	Men finished	Men started
WC	08-09-2013	MontTremblant	29	35	38	50
WC	09-09-2012	MontTremblant	29	33	38	51
WC	11-09-2011	MontTremblant	25	28	39	46
WC	13-10-2010	MontTremblant	28	20	36	42
WC	14-11-2009	MontTremblant	27	42	65	81
WC	08-10-2008	MontTremblant	28	48	47	64
WC	10-10-2007	MontTremblant	25	40	38	55
WC	11-11-2006	MontTremblant	24	35	26	45
CC	20-01-2013	Auckland	16	21	27	38
CC	03-02-2013	Panama	9	13	18	25
CC	12-02-2012	Panama	15	21	18	22
CC	04-05-2013	St. George	27	42	36	53
CC	11-08-2013	Wiesbaden	16	24	34	51
CC	12-08-2012	Wiesbaden	16	17	27	34
CC	14-08-2011	Wiesbaden	19	19	16	23
CC	15-08-2010	Wiesbaden	13	16	17	18
CC	10-08-2008	Wiesbaden	8	9	8	9
CC	19-08-2007	Wiesbaden	11	13	15	17

**Table 3:** **Number of male and female pro finishers in Ironman races from 2003 to 2013, WC = World Championship, CC = Continental Championship**

Race	Date	Location	Women finished	Women started	Men finished	Men started
WC	12-10-2013	Kailua-Kona	27	35	41	53
WC	13-10-2012	Kailua-Kona	26	31	39	53
WC	08-10-2011	Kailua-Kona	26	33	35	51
WC	09-10-2010	Kailua-Kona	40	53	55	68
WC	10-10-2009	Kailua-Kona	38	53	77	101
WC	11-10-2008	Kailua-Kona	42	57	68	99
WC	13-10-2007	Kailua-Kona	43	51	67	91
WC	21-10-2006	Kailua-Kona	48	58	70	91
WC	15-10-2005	Kailua-Kona	44	56	74	87
WC	16-10-2004	Kailua-Kona	35	54	56	85
WC	18-10-2003	Kailua-Kona	33	47	67	89
CC	24-03-2013	Melbourne	22	30	26	46
CC	25-03-2012	Melbourne	18	21	34	40
CC	07-07-2013	Frankfurt	24	30	45	63
CC	08-07-2012	Frankfurt	12	18	25	39
CC	24-07-2011	Frankfurt	14	21	27	41
CC	04-07-2010	Frankfurt	11	13	14	22
CC	05-04-2009	Frankfurt	7	11	12	15
CC	06-07-2008	Frankfurt	9	11	12	17
CC	01-07-2007	Frankfurt	10	12	15	19
CC	23-07-2006	Frankfurt	8	10	13	15
CC	10-07-2005	Frankfurt	7	8	14	14
CC	18-08-2013	MontTremblant	17	19	19	22
CC	19-08-2012	MontTremblant	5	9	8	12

In order to determine the age of each athlete in each race, the date of birth of all the recorded athletes was searched, either through internet search or through direct contact with the athletes or their national federation. A total of 15–20 sets of data had to be deleted due to missing or wrong split times or inability to find the date of birth of an athlete. To determine the age of peak triathlon performance, the top ten women and men ever between 2003 and 2013 for the three different distances were determined and further analysed. In order to determine potential changes across years in the age and in split times and overall race times, the annual top ten women and men were determined and the changes in their age and both split and race times were analysed.

### Statistical analysis

Each set of data was tested for normal distribution (D’Agostino and Pearson omnibus normality test) and for homogeneity of variance (Levene’s test) prior to statistical analyses. Uni- and multivariate regression analyses were used to investigate potential changes in performance and age of the finishers across years. A hierarchical multivariate regression model was used to avoid the impact of a cluster-effect on the results where a particular athlete finished more than once in the annual top ten. Regression analyses of performance were also corrected for the age of athletes to prevent a misinterpretation of the ‘age-effect’ as a ‘time-effect’. Since the change in performance and sex difference in endurance performance is assumed to be non-linear (Reinboud 2004), we calculated the non-linear regression model that fits the data best and compared the linear to the best-fit non-linear model using Akaike’s Information Criteria (AIC) and F-test to show which model would be the most appropriate to explain the trend of the data. In the text, we inserted the model that best explains the data. The ages of the top ten men and women ever between 2003 and 2013 of the different race distances were compared using one-way analysis of variance (ANOVA) with subsequent Tukey-Kramer post hoc analysis. Statistical analyses were performed using IBM SPSS Statistics (Version 22, IBM SPSS, Chicago, IL, USA), CurveExpert Professional (Version 2.0.3, Hyams D.G.) and GraphPad Prism (Version 6.01, GraphPad Software, La Jolla, CA, USA). Statistical significance was accepted with *p* <0.05 (two-sided for *t*-tests). Data in the text and figures are given as mean ± standard deviation (SD).

## Results

### The age of peak triathlon performance in women and men

**Figure 1:**
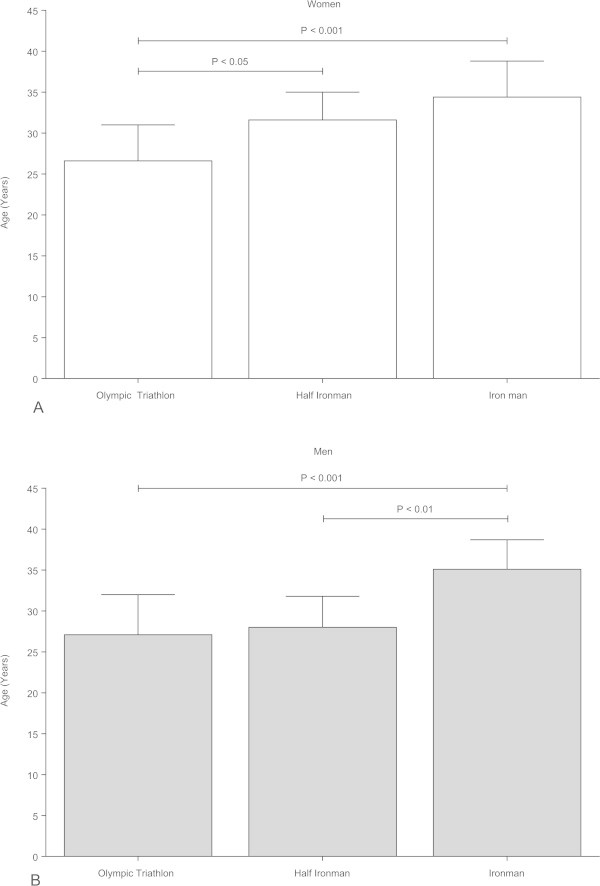
**Age of the overall top ten women (Panel A) and men (Panel B) for the Olympic, the Half-Ironman and the Ironman distance.** The ten fastest athletes during the investigated period of time were included. Results are presented as mean±SD after one-way ANOVA.

The ten fastest women between 2003 and 2013 achieved peak triathlon performance in the Olympic distance at the age of 26.6 ± 4.4 years, in the Half-Ironman distance at 31.6 ± 3.4 years and in the Ironman distance at 34.4 ± 4.4 years (Figure 1A). The age of peak triathlon performance was significantly lower in athletes competing in the Olympic distance compared to athletes competing in the Half-Ironman (*p* < 0.05) and the Ironman distance (*p* < 0.05) triathlon. Between the Half-Ironman and the Ironman distance, there was no significant difference in the age of peak triathlon performance. For men, the ten fastest finishers between 2003 and 2013 achieved their peak triathlon performance in the Olympic distance at the age of 27.1 ± 4.9 years, in the Half-Ironman distance at 28.0 ± 3.8 years and in the Ironman distance at 35.1 ± 3.6 years (Figure 1B). For the Ironman distance, the age of peak triathlon performance was significantly higher compared to the Olympic (*p* < 0.05) and the Half-Ironman distance (*p* < 0.05) triathlon. In male athletes, the age of peak triathlon performance in the Half-Ironman distance was not significantly higher than in the Olympic distance triathlon.

### Age trends across years

**Figure 2:**
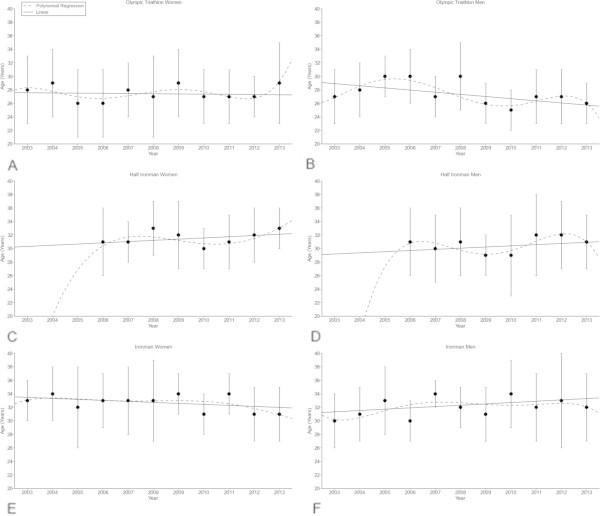
**Age of the annual top ten women (Panel A) and men (Panel B) for the Olympic distance, the annual top ten women (Panel C) and men (Panel D) for the Half-Ironman distance and the annual top ten women (Panel E) and men (Panel F) for the Ironman distance.** Results are presented as mean±SD after linear and non-linear regression analysis.

In the Olympic distance, the age of the annual top ten athletes remained unchanged in women at 27 ± 5 years (Figure 2A) over the last decade but decreased linearly in men (Table 4) from 27 ± 4 (2003) to 26 ± 3 (2013) years (Figure 2B) (*p* < 0.05), with some fluctuation across years. In the Half-Ironman distance, the age of peak triathlon performance remained unchanged (Table 4) at 32 ± 4 years in women (Figure 2C) and 31 ± 5 years in men (Figure 2D). In the Ironman distance, the age of peak triathlon performance for the annual top ten women and men remained unchanged (Table 4) in men at 32 ± 4 years (Figure 2E) and in women at 33 ± 4 years (Figure 2F).

**Table 4:** **Hierarchical multivariate regression analyses for change in age across years for the annual ten fastest women and men with correction for multiple participations for Olympic distance triathlon, Half Ironman and Ironman**

		***β***	SE (***β***)	Stand.***β***	T	***P***
**Olympic distance**	**women**	0.004	0.145	0.002	0.025	0.980
	**men**	-0.260	0.116	-0.211	-2.242	0.027
**Half Ironman**	**women**	0.135	0.186	0.082	0.723	0.472
	**men**	0.119	0.242	0.056	0.492	0.624
**Ironman**	**women**	-0.182	0.127	-0.137	-1.436	0.154
	**men**	0.186	0.130	0.136	1.429	0.156

### Performance trends across years

**Figure 3:**
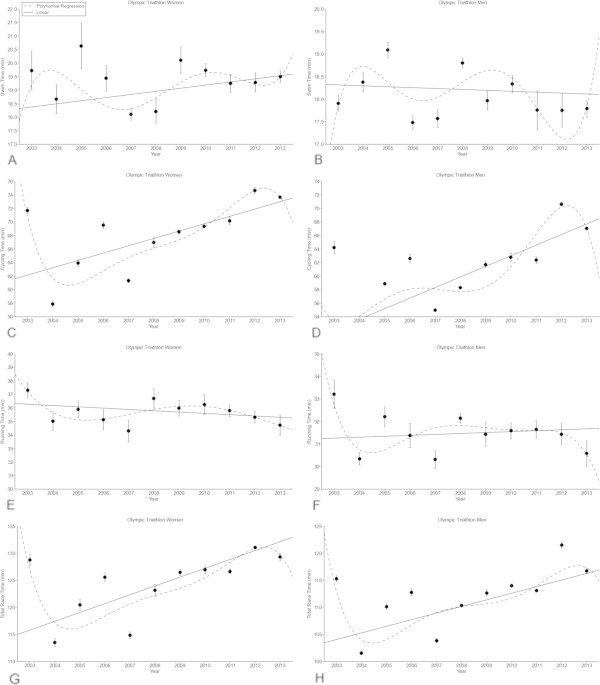
**Swimming split times of the annual top ten women (Panel A) and men (Panel B), cycling split times of the annual top ten women (Panel C) and men (Panel D), running split times of the annual top ten women (Panel E) and men (Panel F) and overall race times of the annual top ten women (Panel G) and men (Panel H) for the Olympic distance.** Results are presented as mean ± SD after linear and non-linear regression analysis.

For the annual top ten women in Olympic distance triathlon, swimming split times remained stable at 19.3 ± 0.5 min (Figure 3A) over the last decade, whereas swimming split times decreased linearly (Table 5) in the annual top ten men from 17.9 ± 0.2 min (2003) to 17.8 ± 0.2 min (2013) (Figure 3B) (*p* < 0.05). Cycling split times increased linearly (Table 5) from 71.7 ± 0.5 min to 73.7 ± 0.8 min in women (Figure 3C) (*p* < 0.05) and from 64.2 ± 1.0 min to 67.1 ± 0.2 min in men (Figure 3D) (*p* < 0.05). Running split times decreased linearly (Table 5) from 37.3 ± 0.6 min to 34.7 ± 0.75 min in women (Figure 3E) (*p* < 0.05) and from 33.2 ± 0.6 min to 30.6 ± 0.6 min in men (Figure 3F) (*p* < 0.05). Overall race time increased linearly (Table 5) from 128.8 ± 1.0 min to 129.3 ± 0.8 min in women (Figure 3G) (*p* < 0.05) and from 115.3 ± 0.6 min to 116.8 ± 0.5 min in men (Figure 3H) (*p* < 0.05).

**Table 5:** **Hierarchical multivariate regression analyses for change in split and overall race times across years for the annual ten fastest women and men with correction for multiple participations and for age for Olympic distance triathlon**

		***β***	SE (***β***)	Stand.***β***	T	***P***
Split time swimming	**women**	-0.002	0.026	-0.007	-0.069	0.945
	**men**	-0.039	0.016	-0.227	-2.410	0.018
Split time cycling	**women**	0.931	0.120	0.599	7.735	<0.0001
	**men**	0.978	0.128	0.600	7.665	<0.0001
Split time running	**women**	-0.073	0.032	-0.219	-2.317	0.022
	**men**	-0.078	0.028	-0.265	-2.789	0.006
Overall race time	**women**	0.966	0.141	0.551	6.831	<0.0001
	**men**	0.967	0.137	0.572	7.081	<0.0001

**Figure 4:**
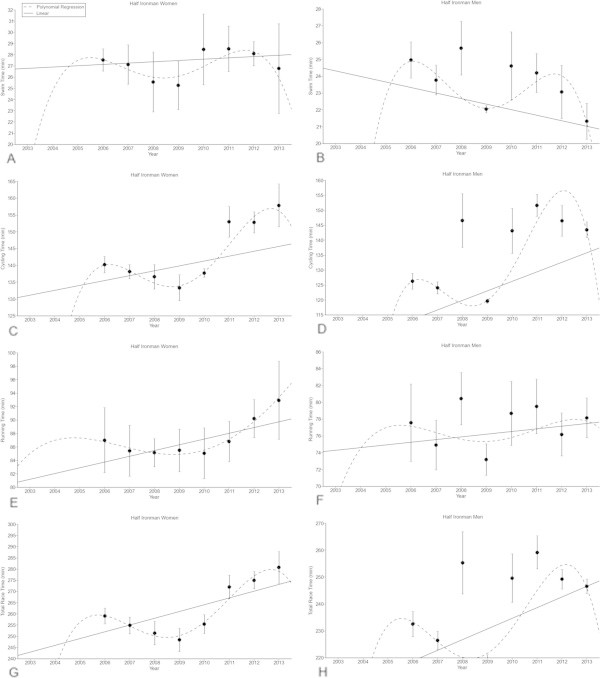
**Swimming split times of the annual top ten women (Panel A) and men (Panel B), cycling split times of the annual top ten women (Panel C) and men (Panel D), running split times of the annual top ten women (Panel E) and men (Panel F) and overall race times of the annual top ten women (Panel G) and men (Panel H) for the Half-Ironman distance.** Results are presented as mean ± SD after linear and non-linear regression analysis.

For the annual ten fastest women in Half-Ironman distance triathlon, swimming split times remained stable at 27.2 ± 2.2 min (Figure 4A) from 2006 to 2013, whereas swimming split times decreased linearly (Table 6) in men from 25.0 ± 1.0 min to 21.3 ± 1.0 min (Figure 4B) (*p* < 0.05). Cycling split times increased non-linearly (*i.e.* polynomial regression 7^th^ degree) in women from 140.2 ± 2.5 min to 157.8 ± 6.3 min (Figure 4C) (*p* < 0.05) whereas in men, cycling split times increased linearly (Table 6) from 126.3 ± 2.7 min to 143.5 ± 2.6 min (Figure 4D) (*p* < 0.05). Running split times increased non-linearly (*i.e.* polynomial regression 7^th^ degree) in women from 87.0 ± 4.8 min to 92.9 ± 5.8 min (Figure 4E) (*p*<0.05) while running split times remained unchanged (Table 6) at 77.3 ± 3.1 min for the annual ten fastest men (Figure 4F). Overall race times increased non-linearly (*i.e.* polynomial regression 7^th^ degree) (Table 6) from 259.1 ± 3.5 min to 280.8 ± 7.0 min in women (Figure 4G) (*p* < 0.05) whereas in men, overall race time increased linearly (Table 5) from 232.5 ± 4.7 min to 246.6 ± 2.7 min (Figure 4H) (*p* < 0.05).

**Table 6:** **Hierarchical multivariate regression analyses for change in split and overall race times across years for the annual ten fastest women and men with correction for multiple participations and for age for Half Ironman triathlon**

		***β***	SE (***β***)	Stand.***β***	T	***P***
Split time swimming	**women**	0.151	0.126	0.134	1.198	0.235
	**men**	-0.370	0.084	-0.450	-4.390	<0.0001
Split time cycling	**women**	2.973	0.321	0.726	9.249	<0.0001
	**men**	3.211	0.506	0.587	6.346	<0.0001
Split time running	**women**	0.812	0.202	0.413	4.025	<0.0001
	**men**	0.159	0.189	0.096	0.840	0.404
Overall race time	**women**	3.823	0.420	0.718	9.108	<0.0001
	**men**	3.019	0.664	0.462	4.548	<0.0001

**Figure 5:**
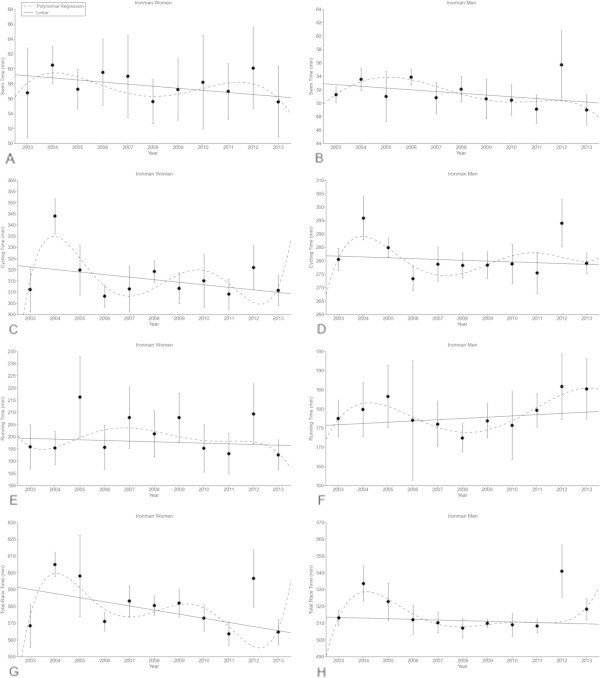
**Swimming split times of the annual top ten women (Panel A) and men (Panel B), cycling split times of the annual top ten women (Panel C) and men (Panel D), running split times of the annual top ten women (Panel E) and men (Panel F) and overall race times of the annual top ten women (Panel G) and men (Panel H) for the Ironman distance.** Results are presented as mean ± SD after linear and non-linear regression analysis.

For Ironman triathletes, swimming split times remained unchanged between 2003 and 2013 (Table 7) at 57.9 ± 4.4 min in women (Figure 5A) and at 51.6 ± 2.4 min in men (Figure 5B). Cycling split times decreased linearly (Table 7) from 311.2 ± 9.9 min to 310.8 ± 6.9 min in women (Figure 5C) (*p* < 0.05), while they remained unchanged (Table 7) at 281.6 ± 5.9 min in men (Figure 5D). Running split times remained unchanged (Table 7) at 201.0 ± 10.0 min in women (Figure 5E) and at 179.0 ± 7.2 min in men (Figure 5F) (*p* < 0.05). Overall race times decreased linearly (Table 7) from 568.4 ± 12.9 min to 564.6 ± 7.6 min in women (Figure 5G) (*p* < 0.05) but remained unchanged in men at 516.8 ± 7.5 min (Figure 5H).

**Table 7:** **Hierarchical multivariate regression analyses for change in split and overall race times across years for the annual ten fastest women and men with correction for multiple participations and for age for Ironman triathlon**

		***β***	SE (***β***)	Stand.***β***	T	***P***
Split time swimming	**women**	-0.098	0.143	-0.067	-0.687	0.494
	**men**	-0.140	0.098	-0.139	-1.435	0.154
Split time cycling	**women**	-1.013	0.379	-0.252	-2.675	0.009
	**men**	-0.336	0.275	-0.118	-1.220	0.225
Split time running	**women**	-0.358	0.382	-0.090	-0.937	0.351
	**men**	0.443	0.259	0.164	1.711	0.090
Overall race time	**women**	-1.440	0.520	-0.261	-2.768	0.007
	**men**	0.010	0.410	0.002	0.025	0.980

## Discussion

This study intended (*i*) to determine the age of peak triathlon performance in world class athletes competing in races held in Olympic, Half-Ironman and Ironman distance triathlons and (*ii*) to investigate a potential change across years in the age of peak triathlon performance of the annual fastest athletes. It was hypothesized that (*i*) the age of peak triathlon performance would be the lowest for the Olympic distance and would be higher in the longer race distances (*i.e.* Half-Ironman and Ironman distance) and (*ii*) the age of peak triathlon performance would increase across years in athletes competing in the Ironman distance, but not for the Olympic and the Half-Ironman distance. The main findings were, (*i*), for the ten fastest women, the age of peak triathlon performance was significantly higher in athletes competing in the Half-Ironman and Ironman distance compared to athletes competing in the Olympic distance, (*ii*), in the ten fastest men, the age of peak triathlon performance was significantly higher in athletes competing in the Ironman compared to athletes competing in the Olympic and the Half-Ironman distance. There was no difference in the age of peak triathlon performance between the Olympic and the Half-Ironman distance in men and between the Half-Ironman and Ironman distance in women. And, (*iii*), the age of the annual ten fastest women and men competing in the Half-Ironman distance and the Ironman distance remained unchanged. In the Olympic distance triathlon, the age of the annual ten fastest women remained unchanged, while it decreased linearly in men, with some fluctuation over the studied period.

### The best triathletes were older in the longer race distances

Recent studies showed that the decline of performance in triathletes competing at national and world class level started earlier in the Olympic distance triathlon than in the longer triathlon race distances (Knechtle et al. 2012b; Lepers et al. 2010). Therefore, we hypothesized that the age of peak triathlon performance would be lower in athletes competing in the Olympic distance compared to the longer triathlon distances (*i.e.* Half-Ironman and Ironman distance). As hypothesized, the age of peak triathlon performance was ~26-27 years in both women and men competing in the Olympic distance triathlons. In women, the age of peak triathlon performance in the Olympic distance was significantly lower than in the Half-Ironman and the Ironman distance and in men significantly lower than in the Ironman distance.

Previous studies showed that women competing at national level in a Half-Ironman triathlon achieved their peak triathlon performance at the age of ~25-39 years and men at ~18-39 years (Knechtle et al. 2012b). These findings suggest that women would achieve peak triathlon performance in Half-Ironman at a higher age compared to men. Consistent with these findings, the age of peak triathlon performance in the Half-Ironman distance was ~32 years in women and ~28 years in men. In women, the age of peak triathlon performance was significantly higher than in the Olympic distance and in men significantly lower than in the Ironman distance.

Studies investigating the age of peak triathlon performance in longer distances than the Ironman distance showed that the mean age of the winning male athletes at international level in a Triple Iron ultra-triathlon distance was ~36 years and the mean age of the winning male athletes at international level in a Deca Iron ultra-triathlon distance was ~38 years (Knechtle et al. 2012a). We postulated that the age of peak triathlon performance in the Ironman distance would be in between the age of peak triathlon performance of the Half-Ironman distance and the longer distances. Consistent with our hypothesis and slightly higher than previous findings (Rüst et al. 2012b; Stiefel et al. 2013a), the present data showed that both female and male Ironman triathletes reached their peak triathlon performance at the age of ~34-35 years. In men, this was significantly higher than the age of peak triathlon performance in the Half-Ironman and the Olympic distance. In women, the age of peak triathlon performance was significantly higher than in the Olympic distance. These findings confirm the hypothesis that the age of peak triathlon performance is higher in longer race distances (*i.e.* Ironman distance).

The higher age of peak endurance performance in longer race distances has also been shown before for a series of different sports, such as ultra-marathon running (Hoffman and Wegelin 2009; Hoffman 2010, Knechtle et al. 2010a, [c]; 2012b, [c]), ultra-cycling (Zingg et al. 2013) and ultra-swimming (Eichenberger et al. 2012). The best performance in marathon running was obtained at the age of ~27 years in men and ~29 years in women (Lara et al. 2014). In comparison, the best performance in a 100-km ultra-marathon was obtained by older runners between the age of 30–54 years for both women and men (Knechtle et al. 2010a [c]; 2012b, [c]). Male ultra-marathoners competing in the age group 30–39 years in a 161-km ultra-marathon achieved the fastest race times compared to athletes competing in the other age groups (Hoffman 2010). In another study investigating 161-km ultra-marathoners, the performance of the athletes in the age group 40–49 years was not different from the performance of the athletes in the age groups <30 years and 30–39 years (Hoffman and Wegelin 2009).

Master athletes being able to maintain their running performance with only a moderate decline as they age and the large number of successful master athletes suggests that master athletes are able to maintain endurance performance with increasing age (Stiefel et al. 2012, Young and Starkes 2005). A recent study showed that the age of peak ultra-marathon performance was higher in the longer distances (*i.e.* 1,000-mile event and 3,100-mile event) (Zingg et al. 2014b). The age of the annual ten fastest women and men in a 50-mile event was ~35 years and increased to the age of ~55 years in men competing in a 1,000-mile event and to the age of ~52 years in women competing in a 3,100-mile event (Zingg et al. 2014b). Findings in ultra-cycling were comparable to findings in ultra-running with the age of peak ultra-cycling performance being at ~36-38 years in the 720-km ‘Swiss Cycling Marathon’ (Zingg et al. 2013). The age of peak swimming speed of ~25 years in 25-km open-water ultra-distance swimming (Zingg et al. 2014a) is higher compared to ~20-23 years in freestyle swimmers competing in 50-m to 1,500-m pool swimming (Rüst et al. 2014). In the open-water ultra-swim race ‘Zürich 12-h swim’ , the best performance was achieved by athletes in the age groups 30–39 years and 40–49 years for both sexes (Eichenberger et al. 2012).

Different factors are responsible for the age-related decline in endurance performance. A progressive reduction in both maximal oxygen consumption (VO_2_max) and lactate threshold appear to be the primary mechanisms associated with the general decline of endurance performance with increasing age (Tanaka and Seals 2008). Considerable atrophy of the skeletal muscles mainly occurs after the age of ~50 years (Faulkner et al. 2007) and it seems that the loss of muscle fibres can be compensated by a hypertrophy of the remaining fibres (Pollock et al. 1997). Physiological factors like VO_2_max and lactate threshold can be regulated by changing both intensity and volume of the training in master athletes (Tanaka and Seals 2008). The training status of master athletes is an important modulator for the decline of performance with advancing age, and changes in VO_2_max and lactate threshold as well as running performance with age are closely related to the amount of distance run in training (Trappe 2007).

Previous experience in a shorter race has been reported as an important predictor for the performance in a longer race in triathlon and in ultra-marathon running (Gulbin and Gaffney 1999; Knechtle et al. 2011b, [c]; Rüst et al. 2011, 2012b). For triathlon, Gulbin and Gaffney (1999) described that previous best performances in Olympic distance triathlons and training distance rather than training pace could partially predict Ironman race time in male and female triathletes. The personal best times in an Olympic distance race and a marathon were strong predictor variables for Ironman race time in both male (Rüst et al. 2011) and female (Rüst et al. 2012b) triathletes. Also in ultra-marathon running, the personal marathon best time was a strong predictor for performance (Knechtle et al. 2011b, [c]).

Master athletes competing in triathlon races improved their performance over the past years (Lepers et al. 2013b; Stiefel et al. 2012) most probably due to advancements in training quality and a better access to these improved training possibilities compared to earlier master athletes (Reaburn and Dascombe 2008; Stiefel et al. 2012). One of the reasons for the higher age in elite triathletes in longer race distances might be pre-race experience, especially pace judgement and both nutritional and race strategies (Abbiss et al. 2006; Gallmann et al. 2014; Whyte 2014). In other endurance disciplines such as ultra-cycling, a high speed in training and appropriate nutrition during the race and not anthropometric characteristics such as a low body mass or low body fat were related to race times (Knechtle et al. 2011a). It was postulated that a reason for the higher age of peak triathlon performance in elite Ironman triathletes compared to elite marathon runners could be the increasing importance of experience in multi-sports disciplines compared to single-sport disciplines (Knechtle et al. 2010b). Previous experience such as the personal best time in a shorter race seemed to be a better predictor for endurance and ultra-endurance athletes than anthropometric and training characteristics (Knechtle 2014). The increase in the age of peak triathlon performance is also influenced by higher participation rates of master athletes and an increase in the competitive spirit in older age groups (Gallmann et al. 2014; Lepers 2008). Also, the increased popularity of Ironman triathlon attracted more master athletes in recent years (Lepers et al. 2013b; Stiefel et al. 2012). For the successful completion of an Ironman triathlon a lot of mental strength and motivation is required and mental strength possibly increases with age in some individuals (Gallmann et al. 2014; Parry et al. 2011).

In recent years there has been an improvement of triathlon performance of master athletes, while the performances of athletes younger than ~40 years remained quite stable (Etter et al. 2013; Lepers et al. 2013b; Stiefel et al. 2012). For example, in ‘Ironman Hawaii’, men older than ~44 years and women older than ~40 years significantly improved their performance in both the split disciplines and in overall race time (Lepers et al. 2013b). Also the age of the annual top ten women and men in ‘Ironman Hawaii’ increased over the last three decades and their performance improved while younger athletes seemed to have reached limits in their Ironman triathlon performance (Gallmann et al. 2014; Lepers et al. 2013b). Therefore, we postulated that the age of the annual top ten athletes over the Ironman distance would increase for both women and men during the studied period. However, our findings showed no change in the age of the annual ten fastest athletes in the Half-Ironman and the Ironman distance during the last decade for both women and men. This might be due to the relatively short period of time of only one decade compared to other studies investigating the age of the annual top ten athletes over a period of ~30 years (Gallmann et al. 2014; Lepers 2008). Gallmann et al. (2014) and Lepers (2008) showed a significant increase in the age of the annual ten fastest finishers in the ‘Ironman Hawaii’ from 1983 to 2012 and 1981 to 2007 respectively. It also has to be considered that there are differences between the ‘Ironman Hawaii’ and its qualifier races regarding the representation of different age-groups (Stiefel et al. 2013b). The only change during the studied period was a decrease in the age of the top ten annual male athletes competing in the Olympic distance from ~27 to ~26 years. Competing in shorter distances requires more strength and speed than it requires endurance and experience, and since those features peak at an earlier age, the age of peak performance is generally lower over shorter distances (Schulz and Curnow 1988). We assume that several factors may have led younger athletes to compete in the Olympic distance. For example the offered prize money and the perspective to compete in the Olympic Games. The slight decrease in the age of the annual top ten athletes might also be influenced by the highly competitive level for professional athletes competing in the Olympic distance triathlon.

### Improvements in split and overall race times across years

Regarding the performance over the last decade in the split times for the three distances, athletes improved in several split disciplines where men improved their running and swimming split times and women improved their running split times in the Olympic distance triathlon over the last decade. This might be because Olympic distance triathletes invested more time in running training to improve overall race performance, as it has been seen in Ironman triathletes (Rüst et al. 2012a). In the Half-Ironman distance, swimming split times improved in men over the studied period. This could be due to technological advances in wetsuits, but it will not explain why women were not able to improve their swimming split times during the same period. The only changes during the last decade over the Ironman distance were an improvement in cycling split times and overall race times in women. The improvement in overall race time is most likely due to the improvement in cycling split times. It has been shown for ultra-triathletes that both cycling and running split times but not swimming split times were associated with overall race times (Knechtle et al. 2007). Therefore, it can be assumed that Ironman triathletes invested more time in training to improve in the split disciplines with more importance for the race (Rüst et al. 2012a). The decrease in cycling split times might also be due to improvement in equipment (Bentley et al. 2002), but that will not explain why male triathletes were not able to improve their cycling split times as well.

### Limitations, strengths and implications for future research

The weakness of this study is the relatively short studied period compared to similar studies. In contrast to other studies on the subject of the age of peak triathlon performance, the strength of this study is the direct comparison between the three different distances rather than focussing on solely one race distance. Regarding the study design, a limitation in this retrospective study is the fact that we were unable to consider factors of endurance performance such as physiological (Saunders et al. 2004) and anthropometric parameters (Knechtle et al. 2010d), training intensity (Knechtle et al. 2010d), previous experience (Knechtle et al. 2010b), motivation (Houston et al. 2011), and environmental conditions of the race (El Helou et al. 2012; Ely et al. 2007). Despite these limitations this study reveals beneficial information to athletes and coaches and expands the existing data about the exact age of peak triathlon performance. In future studies data about pre-race experience as well as training volume should be additionally collected to determine the impact of these factors on race performance and the age of peak triathlon performance.

### Practical applications for athletes and coaches

This study shows that the age of the fastest race times were higher in the longer triathlon distances. While the fastest athletes were ~26-27 years in the Olympic distance triathlon, the age of the fastest triathletes was ~28-31 years in the Half-Ironman and ~34-35 years in the full Ironman distance triathlons. Athletes and coaches can now better plan the career of a triathlete who is intending to compete in Ironman triathlon at world class level. Since personal best time in an Olympic distance triathlon is a strong predictor for Ironman race time, athletes may compete until the age of < 28 years in Olympic distance, and then switch from the age of ~28-31 years to the Half-Ironman to change then to the full Ironman distance.

## Conclusions

In summary, the age of peak triathlon performance was higher in the Ironman distance than in the Olympic distance triathlon for both women and men. Also the age of peak performance in male triathletes was higher in the Ironman distance than in the Half-Ironman distance and it was higher in the Half-Ironman distance than in the Olympic distance triathlon in women. The age of the annual top ten male and female athletes remained stable over the last decade for the three different distances, except for a slight decrease in the age of male triathletes competing in the Olympic distance triathlon. In the Olympic distance triathlon, the fastest male and female triathletes were at the age of 26–27 years. The fastest men were ~28 years old in the Half-Ironman distance and ~35 years in the Ironman distance. In women, the fastest athletes were ~31.5 years old in the Half-Ironman distance and 34.5 years in the Ironman distance. The results of the present study may contribute to a more precise career planning for both coaches and athletes in order to determine the right point in time to switch from the shorter to the longer distances. The switch from the Olympic distance to the Half-Ironman distance and/or the Ironman distance can be planned more accurately, as with the right timing a top athlete over the Olympic distance manages to stay at the top after changing the racing distance.
